# A systematic review of non-standard dosing of oral anticancer therapies

**DOI:** 10.1186/s12885-018-5066-2

**Published:** 2018-11-22

**Authors:** Faouzi Djebbari, Nicola Stoner, Verna Teresa Lavender

**Affiliations:** 1 Oxford Cancer and Haematology Centre & NIHR Oxford Biomedical Research Centre, Churchill Hospital, Oxford University Hospitals NHS Foundation Trust, Old Road, Headington, Oxford, OX3 7LE UK; 2 Oxford Cancer and Haematology Centre & Oxford Cancer Research Centre, Churchill Hospital, Oxford University Hospitals NHS Foundation Trust, Old Road, Headington, Oxford, OX3 7LE UK; 3 Faculty of Health and Life Sciences, Oxford Brookes University, Marston Road, Oxford, OX3 0FL UK

**Keywords:** Systemic anticancer therapy, SACT, Chemotherapy, Cytotoxic, Targeted therapy, Oral, Non-standard, Prescribing, Dose, Review

## Abstract

**Background:**

The use of oral systemic anticancer therapies (SACT) has increased and led to improved cancer survival outcomes, particularly with the introduction of small molecule targeted agents and immunomodulators. Oral targeted SACT are, however, associated with toxicities, which might result in reduced quality of life and non-adherence. To reduce treatment-related toxicity, the practice of non-standard dosing is increasing; however guidance to govern this practice is limited. A systematic review was conducted to identify evidence of, and outcomes from, non-standard dosing of oral SACT in oncology and malignant haematology.

**Methods:**

A comprehensive search of 78 oral SACT was conducted in the following databases: MEDLINE®, EMBASE®, Cochrane Library©, and Cumulative Index to Nursing and Allied Health Literature (CINAHL©). Studies were selected based on predefined inclusion/exclusion criteria, and were critically appraised. Extracted data were tabulated to summarise key findings. Due to diversity of study designs and heterogeneity of reported outcomes, studies were categorised and evidence was synthesised in three main themes: dose interruption; dose reduction; and other dosing strategies.

**Results:**

Thirty-four studies were eligible for inclusion: four clinical trials, fifteen cohort studies and fifteen case reports. Evidence for non-standard dosing was reported for eleven oral SACT. Dose interruptions were the most commonly reported strategy (14 studies); nine studies reported dose reductions; and eleven reported other dosing strategies. Eight retrospective cohort studies reported dose interruption of sunitinib in renal cell carcinoma and showed either similar or improved responses and survival outcomes, and fewer or equivalent high grade toxicities, compared to the standard schedule. Four cohort studies retrospectively evaluated dose reductions of imatinib, gefitinib or erlotinib, for chronic myeloid leukaemia and non-small cell lung cancer, respectively. Other dosing strategies included alternate-day dosing. The quality of the evidence was limited by the small sample size in many studies, retrospective study designs, and lack of reported toxicity and/or QoL outcomes.

**Conclusions:**

This review identified limited evidence to support current non-standard dosing strategies, but some of findings, e.g. dose interruption of sunitinib, warrant further investigation in large-scale prospective clinical trials.

**Electronic supplementary material:**

The online version of this article (10.1186/s12885-018-5066-2) contains supplementary material, which is available to authorized users.

## Background

Systemic anticancer treatment (SACT) has undergone a major revolution in the last decade [1]. The recent discovery and approval of multiple oral SACT has led to improved survival outcomes for people with cancer [1]. Oral SACT includes cytotoxic agents (e.g. temozolomide), small molecule targeted agents (e.g. crizotinib), immunomodulators (e.g. lenalidomide) and hormone modulators (e.g. enzalutamide) [2], which have a variety of molecular mechanisms and differing toxicity profiles.

Depending on the licensed dose, some agents are administered daily and continuously until disease progression or unacceptable toxicity, other agents are administered on specific days with a scheduled break within the treatment cycle, and some are administered for a specific treatment duration then discontinued thereafter (e.g. temozolomide) [2]. For instance, the licensed dose of imatinib for chronic myeloid leukaemia (CML) in chronic phase is 400 mg once daily continuously [3] and the licensed dose of sunitinib in metastatic renal cell carcinoma (RCC) is 50 mg once a day for four consecutive weeks followed by a 2 weeks rest period [4].

Many oral SACT are associated with high treatment costs, particularly novel therapies. For instance in the UK, the monthly National Healthcare Service (NHS) indicative prices of sunitinib 50 mg capsules, imatinib 400 mg tablets, and lenalidomide 25 mg capsules, according to the British National Formulary (BNF) are £3138.80, £1946.67 and £4368.00, respectively [2] and the monthly cost of combination therapy for metastatic melanoma (dabrafenib/trametinib) at full dose is £10,400 [2].

Oral SACT are associated with high-grade toxicities that lead to dose reduction, dose interruption/delay, or treatment discontinuation [5]. High-grade toxicities can reduce quality of life (QoL), and subsequent dose interruption or treatment discontinuation may reduce treatment efficacy [5–7]. One approach to maintain patients on continuous SACT is to prescribe non-standard doses, where unlicensed doses/schedules are used to reduce toxicities, improve quality of life (QoL) and extend the duration of therapy.

Governance guidelines are implemented nationally and locally in the UK to ensure evidence-based safe and effective prescribing practice, which is based on robust evidence from large clinical studies and is undertaken in accordance with the Summary of Product Characteristics (SPC) [8]. In the UK, the National Institute for Health and Care Excellence (NICE) assesses evidence to produce up-to-date rigorous guidelines and recommendations on indication, licensing, approvals, and dosing of all oral SACT [9]. Guidance governing non-standard oral SACT doses is, however, either limited or non-existent.

An initial scoping review about non-standard dosing strategies of oral SACT did not identify any published comprehensive reviews on this topic. Yet, case reports and cohort studies investigating these strategies have been published: Dooley et al (2014) reported a case series of 6 melanoma patients managed with dose reductions and/or intermittent dosing of vemurafenib [10]; Popat et al (2014) reported a retrospective cohort study of 39 myeloma patients treated with alternate day dosing of lenalidomide [11].

The purpose of this systematic review was, therefore, to identify evidence of, and outcomes (efficacy, toxicity, QoL) from, non-standard dosing of oral SACT in oncology and malignant haematology, in order to inform prescribing practices. A secondary aim of this review was to inform future research that aims to evaluate the feasibility of oral SACT non-standard dosing practice.

## Method

### Search strategy

The review was conducted following systematic review criteria described by Grant and colleagues (2009) and in accordance with the Preferred Reporting Items for Systematic Review and Meta-Analysis Protocols (PRISMA-P) guidelines [12, 13]. The review protocol was registered on the PROSPERO database (CRD42017076195) and published prior to conducting the review [14]. The protocol paper details the full search strategy [14].

Search terms used were drug names of 78 (all) oral SACT listed in the British National Formulary (2017) [2], relevant Medical Subject Headings (MESH) terms for anticancer agents, and synonyms for non-standard dosing [14]. The list of oral SACT included in the search strategy is presented in Additional file 1: Table S1. The search terms were used with the Boolean operators AND and OR to search MEDLINE®, Embase®, Cochrane Library©, and Cumulative Index to Nursing and Allied Health Literature (CINAHL©) databases [14]. No date restriction was applied, but the search was restricted to English language. The search was completed in September 2017 and was updated in April 2018. The search was expanded using prospective citation chaining in the Web of Science and retrospective snowballing of reference lists of included studies to ensure a sensitive, comprehensive search.

### Screening search results

Search results were independently double-screened by the research team using eligibility and exclusion criteria shown in Table 1, both at abstract and full text screening stages. Disagreements between two researchers were reviewed by a third researcher to reach agreement.

**Table 1:** eligibility criteria

Eligibility Criteria	
Inclusion	Exclusion
•Studies of malignant disease •Studies of patients aged ≥18 years •Studies of oral SACT with non-conventional dosing •Studies examining the prescribing practices using unlicensed (non-standard) doses or schedules of oral SACT •Meta-analysis •Late phase clinical trials •Cohort studies •Cross-sectional studies •Retrospective studies •Observational studies •Case-control studies •Case-reports •MHRA: reports, legislative documents	•Studies of parenteral SACT (e.g. IM, IV, SC, IT) •Studies of oral SACT where non-conventional dosing has been used, but cannot be extracted independently of other reported data •Studies comparing different licensed doses of oral SACT for the same antineoplastic indication •New standard dose-finding studies •Animal studies •Early phase clinical trials •Pharmacokinetic studies •Narrative reviews •Opinion papers •Education papers •Commentaries •Editorials •Conference abstracts

### Quality appraisal and data extraction

Standardised Critical Appraisal Skills Programme (CASP) tools were used to appraise the quality of study design and reporting [15]. CASP tools used were specific for the type of study reviewed (e.g. randomised clinical trial, cohort study, and case report) [15]. Studies were assigned a quality rating of high, moderate to high, moderate, moderate to low, or low. Decisions were made to include lower quality studies where relevant data had been reported; limitations of data reported in lower quality studies was transparently reported in the review. Extracted data were tabulated using pre-defined categories in order to sort and analyse key findings (Table 2).

**Table 2:** Data extraction table

Data to be extracted	Item
Publication ID	• Author • Publication date
Study aim	• Title/Purpose/Aim
Study design	• Study type: meta-analysis, late phase clinical trial, cohort study, cross-sectional study, retrospective study, observational study, Case-control study, case-report • Measurement tools, instruments, measures, outcome criteria
Non-conventional dosing characteristics	• Oral SACT name • Dose • Duration of therapy
Sample characteristics	• Number of participants • Country • Age • Gender • Cancer type
Findings	• Reported efficacy outcomes • Reported side effects/toxicity outcomes • Reported health-related quality of life • Any other findings
Strengths and limitations	• Findings of critical appraisal

### Data extraction

Extracted data was reviewed by all the research team and tabulated to effectively report key findings. Key data extracted from each study were: author and year of publication, aims, design, drug schedule, and reported outcomes (efficacy, toxicity and QoL) (Table 2).

### Data analysis

In view of diversity of study designs, variability in numbers of identified studies per drug, and heterogeneity in reported outcomes from one study to another, studies were categorised into the themes: dose interruption; dose reduction; and other dosing strategies. The research design and characteristics of non-standard dose interventions, clarity of reporting, and statistical significance of reported data, were assessed to determine the strengths and limitations of the evidence base as a whole under each of the above themes. The findings of this analysis are presented below.

## Results

### Search results

**Fig. 1:**
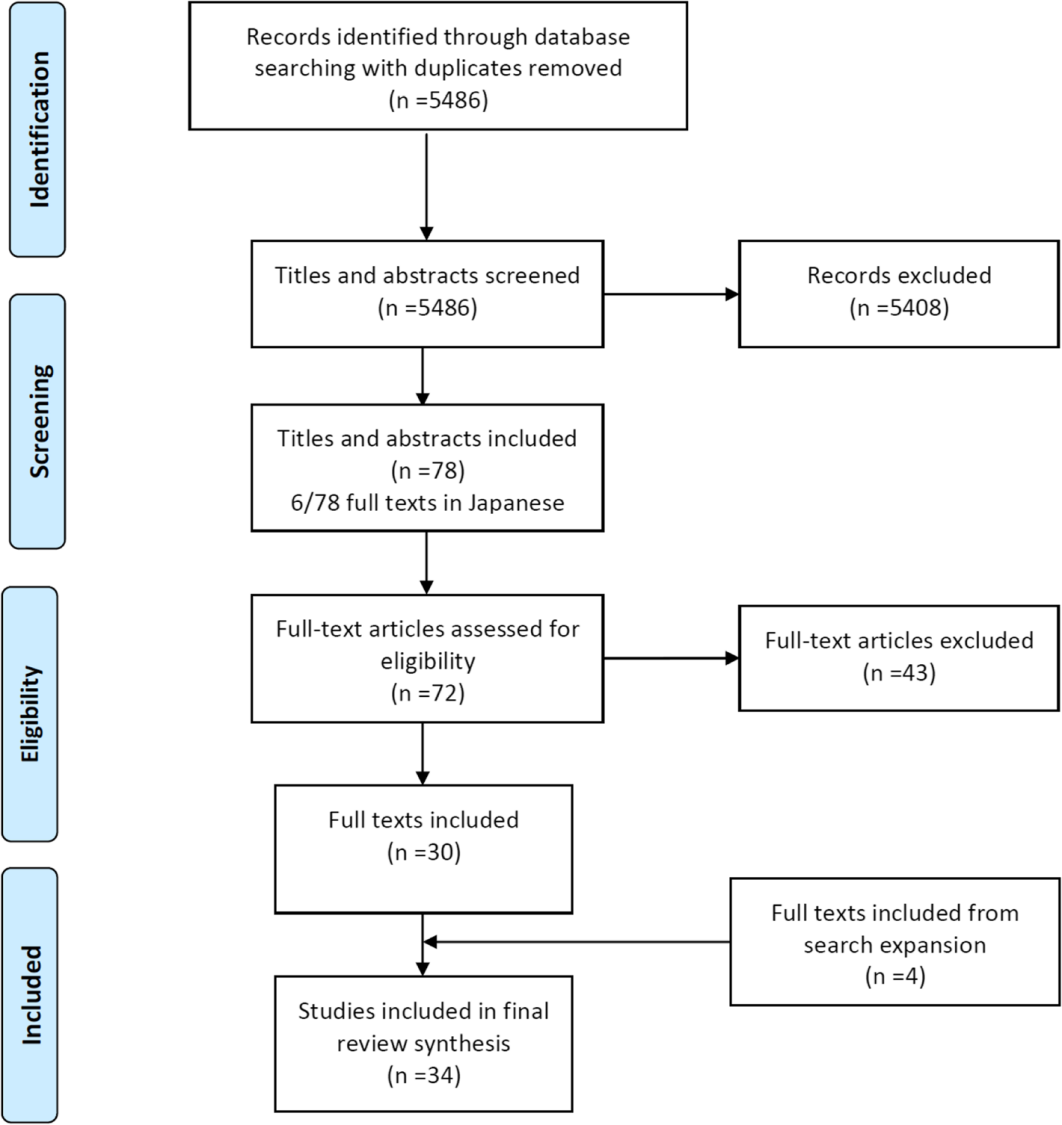
Flow diagram of search strategy and inclusion/exclusion

Of 5486 search results, 31 studies were eligible for inclusion. One study was later excluded because treatment schedules used were not in line with current practice [16]. During the process of search expansion, four additional studies were included [10, 17–19]. In total, 34 studies met eligibility criteria for this review (Fig. 1); 23 reporting non-standard dosing of oral SACT in solid tumours and 11 in haematological malignancies. Four studies were late phase clinical trials [20–23], 15 were cohort studies and 15 were case reports. Non-standard dosing was identified for eleven different oral SACT, as reported in Tables 3-5. The number of studies per drug investigated was as follows: sunitinib (10), imatinib (7), sorafenib (2), vemurafenib (3), dasatinib (2), lenalidomide (2), crizotinib (2), erlotinib (2), gefitinib (2), temozolomide (1) and thalidomide (1). Nine studies were conducted in Italy, eight in Japan, 6 in the USA, three in the UK, three in Germany, two in South Korea, one in each of Brazil, China and Austria. Non-standard dosing strategies reported were dose interruptions, dose reductions and a variety of other strategies. Dose interruption strategies were the most common non-standard dosing strategy described (14 studies), dose reductions (9 papers), and other dosing strategies (11 studies, of which, two reported the use of alternate day dosing of lenalidomide [11, 23]).

**Table 3:** Studies reporting dose interruptions as non-conventional dosing strategy

Publication and country	Aims	Design	Schedule	Reported outcomes		
				Efficacy	Toxicity	QoL
Wick et al (2007) Germany [20]	To evaluate toxicity and efficacy of an alternating weekly regimen of temozolomide	Prospective non-randomized late phase trial, sample = 90, indication: recurrent glioma (64 glioblastoma)	150 mg/m^2^ (days 1–7 and 15-21 every 4/52). Dose reductions/increases in steps of 25-50mg/m^2^. **Licensed monotherapy starting dose: 150 mg/m** ^**2**^ **od 5/7 of a 28 day cycle**	6 month PFS rate in glioblastoma group 43.8%. Median PFS 24 weeks. Median OS from diagnosis of progression 38 weeks.	% per week neutropenia: G2 (7.6), G3(1.1), G4(0.1), Lymphopenia: G2(1.6), G3(1.1), G4(0.7), thrombocytopenia: G2(5.9), G3(8.5), G4(1.9)	Nil
Buti et al (2012) Italy [28]	To investigate the tolerability and efficacy of a modified schedule of sunitinib following ≥G2 toxicities	Retrospective cohort study, sample = 8, indication: metastatic RCC	Starting at 50 mg od 4/52 and 2/52 off. Modified schedule: daily on days 1-5 (weeks 1-5) and od on days 1, 3 and 5 (week 6) **Licensed monotherapy starting dose:50 mg od 28/7 plus 2/52 break**	Median PFS and OS 16.3 and 28.5 months. 70% of patients alive at 2 years.	Toxicity reduced with modified schedule	Nil
Mangiacavalli et al (2012) Italy [21]	To explore efficacy and peripheral neuropathy of low-dose intermittent thalidomide	Late phase randomised trial, sample = 23, indication: multiple myeloma	Arm A (*n* = 13: 100 mg daily days 1-21 plus 7 days break), Arm B (*n* = 10: 100 mg daily continuously) **Licensed starting dose: 200 mg od but it can start lower (50 mg–100 mg)**	Median PFS and OS in arms A and B (7 vs. 42 months, *p* = 0.02), (24 months vs. not reached < 0.001).	Majority of patients (74%) suffered some peripheral neuropathy PN (G1 49%, G2 39%, G3 12%). Median time to PN occurrence 7.5 months. PN incidence in arms A and B (62% vs. 90%, *P* = 0.15).	Nil
Atkinson et al (2013) US [25]	To investigate the impact of alternative schedules of sunitinib on clinical outcomes	Retrospective cohort study, sample = 185, indication: metastatic RCC	Standard subgroup 1 (S1) *n* = 98: 50 mg od 4/52 on 2/52 off. Alternative dosing Subgroup 2 (S2) *n* = 87: 50 mg 2/52 on 1/52 off or other alternative schedule. **Licensed monotherapy starting dose:50 mg od 28/7 plus 2/52 break**	Median PFS in S1 and S2 was 4.3 vs. 14.5 months, *p* < 0.0001), median TOT (4.1 vs. 13.6 months, p < 0.0001), median OS (17.7 vs. 33.0 months, p < 0.0001).	63 patients experienced AE leading to schedule change. Rate of G3–4 fatigue 10%, hand-foot syndrome 8% and diarrhoea 5%. Incidence of AE in S2 < 30%. Incidence reduced on transition from S1 to S2 dose	Nil
Kondo et al (2013) Japan [26]	To compare efficacy and AE profile of an alternative sunitinib schedule with standard schedule	Retrospective cohort study, sample = 48, indication: metastatic RCC	Subgroup 1 (S1) *n* = 22: 50 mg od 4/52 and 2/52 off. Subgroup 2 (S2) *n* = 26: 50 mg od 2/52 and 1/52 off. **Licensed monotherapy starting dose:50 mg od 28/7 plus 2/52 break**	CR and PR rates S1 (5%, 45%) S2 (8%, 24%). Objective response in S1, S2 (50% vs. 32%, *p* = 0.12), median PFS S1 and S2 (9.1 vs. 18.4 months, *p* = 0.13).	No difference in incidence of most AEs between subgroups. More frequent hand–foot syndrome (HFS) and diarrhoea in S1 compared to S2	Nil
Neri et al (2013) Italy [29]	To determine if bi-weekly sunitinib dosing can maintain the same efficacy as standard schedule whilst reducing AE	Retrospective cohort study, sample = (31), indication: metastatic RCC	Alternative schedule: 50 mg od 2/52 and 1/52 off. Dose was reduced to 37.5 mg for ≥ G2 toxicity in 4 patients. **Licensed monotherapy starting dose:50 mg od 28/7 plus 2/52 break**	ORR 42%, CR 10%, PR 32%. Median PFS 16.4 months. Median OS 18.1 months.	Reported as important toxicities: anaemia, gastrointestinal effects, fatigue and hypertension but manageable.	Nil
Ohzeki et al (2014) Japan [30]	To report efficacy and toxicity outcomes of 2 sunitinib schedules: alternative and standard	Retrospective cohort study, sample = 54, indication: metastatic RCC	Standard dose subgroup 1(S1): 4/52 on 2/52 off (daily dose not specified). Alternative dose subgroup 2(S2): any schedule different to standard dose **Licensed monotherapy dose:50 mg od 28/7 plus 2/52 off**	PR or stable disease in S1 and S2 (47.1% vs. 95.5%, *P* < 0.001). Median TTF (4.1 vs. 11.6 months, *p* = 0.04). Median PFS (4.1 vs. 11.3 months, *p* = 0.031). Median OS (12 vs. 32.1 months, *p* = 0.0018).	AE significantly less common in S2, including most high-grade AE.	Nil
Dooley et al (2014) UK [10]	To report efficacy and toxicity outcomes of 6 cases treated with intermittent vemurafenib	Case series, sample = 6, indication: BRAF V600E-mutant melanoma	Variation between 6 cases: range 240 mg–960 mg bd. Schedules: continuous, 2/52 on 2/52 off, 1/52 on 1/52. Different durations/interruptions **Licensed monotherapy starting dose:960 mg bd**	Variation between 6 cases. Response range: stable disease to good response, progression in some cases.	Toxicity outcomes variable depending on case and dose	Nil
Koop et al (2014) Germany [32]	To report efficacy and toxicity outcomes of 1 case treated with intermittent vemurafenib	Case report, sample = 1, indication: metastatic BRAF V600E-mutated melanoma	Dose: 960 mg bd 6/52, off 12/52, on 8/52, off 11/52, on 6/52 **Licensed monotherapy starting dose:960 mg bd**	PR at 6 weeks, mixed response (progression on interruption and response on re-initiation)	acanthoma on the trunk, photosensitivity, loss of taste and fatigue	Nil
Russo et al (2015), Italy [23]	To investigate the effects of a non-standard, intermittent imatinib dose in elderly patients	Late phase trial, sample = 76, indication: CML	Schedule: 1/52 on 1/52 off (weeks 1-4), 2/52 on 2/52(weeks 5-12), 1/12 on 1/12 off thereafter. Daily dose: 400 mg (81%), 200-300 mg (17%), 600 mg (1 patient). **Licensed monotherapy starting dose:400 mg od**	21% lost CCgR and MR3.0, 21% lost MR3.0 alone. No progression recorded. 9 patients died on remission.	Nil	Nil
Bracarda et al (2015) Italy [27]	To evaluate safety and efficacy outcomes of an alternative schedule of sunitinib	Retrospective multicentre cohort study, sample = 460, indication: metastatic RCC	Subgroup 1 (S1) *n* = 208: 50 mg od 4/52 on 2/52 off, switched to 2/52 on 1/52 off. Subgroup 2 (S2) *n* = 41: 50 mg od 2/52 on 1/52 off. External control group (E) *n* = 211: 50 mg od 4/52 on 2/52 off. **Licensed monotherapy starting dose:50 mg od 28/7 plus 2/52 break**	Median PFS in S1, S2 and E (30.2 vs. 10.4 vs. 9.7 months). Median OS (not reached vs. 23.2 vs. 27.8 months).	Incidence of G ≥ 3 in alternative schedule compared to standard (8.2% vs. 45.7%, P < 0.001).	Nil
Pan et al (2015) China [24]	To assess efficacy and tolerability and HRQoL of alternative vs. traditional sunitinib dosing	Retrospective cohort study, sample = 108, indication: metastatic RCC patient	3 subgroups: Subgroup 1(S1) *n* = 50: 50 mg od 4/52 on 2/52 off. Subgroup 2(S2) n = 26: 50 mg od 2/52 on 1/52 off. Subgroup 3 (S3) *n* = 32: starting as per S1 and switched as per S2 **Licensed monotherapy starting dose:50 mg od 28/7 plus 2/52 break**	No difference in tumour response between S1–3. Median PFS S3 vs. S2 vs. S1 (11.2 vs. 9.4 vs. 9.5 months, respectively, *P* = 0.030).	Incidences of diarrhoea, fatigue, hand-foot syndrome, and neutropenia less common in S3 compared to S2 and S1 (*P* < 0.05).	HRQoL better in S3
Miyake et al (2015) Japan [31]	To investigate clinical significance of changing from standard to alternative sunitinib dosing	Retrospective cohort study, sample = 45, indication: metastatic RCC	50 mg od 4/52 on 2/52 off, then changed to 50 mg od 2/52 on 1/52 off **Licensed monotherapy starting dose:50 mg od 28/7 plus 2/52 break**	Nil	Toxicities occurred on both schedules. Statistically higher ≥G3 toxicities with schedule change.	HRQOL Better with reduced dose
Jonasch et al (2018) US [22]	To assess efficacy and toxicity of an alternative schedule of sunitinib	Late phase trial, sample = 59, indication: previously untreated RCC	Stating at Level 0, and reducing to other alternative schedules (Levels −1 to −5) if toxicities: Level 0: 50 mg od 2/52 on 1/52 off **Licensed monotherapy starting dose:50 mg od 28/7 plus 2/52 break**	ORR 56%, CR 2%, PR 54%. Median PFS 13.7 months. Median OS not reached.	G3≥ fatigue, diarrhoea or HFS in 25% of patients. Two discontinuations due to unresolved G3 fatigue and 4 due to CHF, proteinuria, concomitant G2 toxicities or osteonecrosis.	Nil

Licensed doses of individual drugs have been listed in bold, following schedule information, for reader's information

*Abbreviations*: *PFS* Progression-free survival, *OS* Overall survival, *RCC* Renal cell carcinoma, *od* Once daily, *bd* Twice a day, *PN* Peripheral neuropathy, *AE* Adverse events, *TOT* Time on treatment, *CR* Complete response, *PR* Partial response, *HFS* Hand-foot syndrome, *ORR* Overall response rate, *TTF* Time to treatment failure, *CML* Chronic myeloid leukaemia, *CCgR* Complete cytogenetic response, *MR* Molecular response, *HRQoL* Health-related quality of life, *CHF* Congestive heart failure

**Table 4:** Studies reporting dose reductions as non-conventional dosing strategy

Publication and country	Aims	Design	Schedule	Reported outcomes		
				Efficacy	Toxicity	QoL
Binder et al (2010) Germany [34]	To assess efficacy and tolerability outcomes of dose-reduced erlotinib	Retrospective cohort study, sample = 53, indication: advanced NSCLC	Subgroup 1 (S1): n = 31:150 mg od. Subgroup 2 (S2):n = 9: 100 mg od. Subgroup 3 (S3): *n* = 9: 75 mg or 50 mg od **Licensed monotherapy starting dose:150 mg od**	Median TTP months (S1: 3.1, S2: 6.2, S3 18.4). Median TTP among patients with no toxicity vs. with toxicity (1.0 vs. 5.4, P = 0.001).	Toxicity leading to discontinuation (8%). G3 rash (9/53), G2–3 nail toxicity (9/53), G2–3 diarrhoea (4/53), G3 conjunctivitis or keratitis in 2/53	Nil
Breccia et al (2010) Italy [18]	To determine if reduced dosing of imatinib is as effective as standard	Retrospective cohort study, sample = 45, indication: CML	Starting dose 400 mg od, reduced to 300 mg od (43 patients) and 200 mg od (2 patients). **Licensed monotherapy starting dose:400 mg od**	MCyRs in 67% of patients at 6/12 from dose reduction, CCyR 58%, CMR 18%. All patients on MCyR reached CCyR at 12/12. CMR in 20% and MMR 22%.	Nil	Nil
Serpa et al (2010) Brazil [17]	To report in 4 cases the safety and efficacy of low doses of dasatinib	Case report, sample = 4, indication CML	Varied: range 20-140mg od, different durations and interruptions **Licensed monotherapy starting dose:100 mg od**	Responses include CCyR or MMR or both.	High grade thrombocytopenia and neutropenia	Nil
Abbadessa et al (2011) Italy [39]	To report in four cases treated with sorafenib, the efficacy and tolerability of prolonged low dosing	Case report, sample = 4, indication: advanced HCC	Only case 4 has extractable dose data: 400 mg 10/7, withheld for 1/12, restarted at 50% for 4/12, withheld 9/7, restarted 400 mg e.o.d **Licensed monotherapy starting dose:400 mg bd**	PR after 3 months. Stable response at 2 years. CR at 62 months.	G3 hand-foot skin reaction on full dose.	Nil
Hoon Sim et al (2014) S.Korea [35]	To evaluate the effect of gefitinib dose reduction on survival	Retrospective cohort study, sample = 263, indication: EGFR NSCLC	Subgroup 1(S1): n = 240: 250 mg od. Subgroup 2 (S2) n = 23: mean dose intensity index 0.84. **Licensed monotherapy starting dose:250 mg od**	Median PFS S1 vs. S2 (10.8 vs. 14.0 months, *P* = 0.042). Median OS in S1 and S2 (29.6 vs. 54.5 months, *P* = 0.02).	Toxicities leading to dose reduction: skin (5/23), abnormal LFTs (11/23), both toxicities (6/23).	Nil
Jung Sung et al (2014) S.Korea [33]	To evaluate BSA as index in defining appropriate imatinib dosage	Retrospective cohort study, sample = 70, indication: CML	Subgroup 1(S1) *n* = 25: ≥ 400 mg od. Subgroup 2 (S2) *n* = 45: ≤ 300 mg od. **Licensed monotherapy starting dose:400 mg od**	Dose/BSA important index of CCyR12. Higher CCyR12 probability if IM/BSA > 206.7 mg/m^2^.	Toxicities leading to doe reductions: severe neutropenia or thrombocytopenia	Nil
Zipin et al (2014) US [19]	To describe a case of reduced dose of imatinib in terms of efficacy	Case report, sample = 1, indication: CML	400 mg od 4/52, withheld 7/52, restarted 100 mg od. At week 23, increased to 200 mg od 22/52 until date of report **Licensed monotherapy starting dose:400 mg od**	FBC normalise on therapy. CCyR on 200 mg od.	Nil	Nil
Shinoda et al (2015) Japan [36]	To describe a case of reduced doses sorafenib (efficacy and toxicity)	Case report, sample = 1, indication: advanced HCC	Variable dose range: 800 mg od to 200 mg e.o.d **Licensed monotherapy starting dose: 400mg bd**	Good response CT at month 8. No progression on last follow up.	G3 hypertension at 800 mg od.	Nil
Jamison et al (2016) US [37]	To report 2 cases of low dose of dasatinib (efficacy and toxicity)	Case report, sample = 2 indication: CML	Patient 1: 70 mg bd reduced gradually to 20 mg od. Patient 2: 70 mg bd reduced gradually to 50 mg od **Licensed monotherapy starting dose:100 mg od**	BCR-ABL p 210 fusion transcript undetectable. Time to MMR: 9 and 10 months respectively.	Patient 1: grade 2-3 arthralgia, myalgia, and peripheral oedema. Patient 2: painful maculopapular rash and pancreatitis.	Nil

Licensed doses of individual drugs have been listed in bold, following schedule information, for reader's information

*Abbreviations*: *NSCLC* Non-small cell lung cancer, *TTP* Time to progression, *CML* Chronic myeloid leukaemia, *MCyR* Major cytogenetic response, *CCyR* Complete cytogenetic response, *CCyR12* CCyR at 12 months, *crCMR* Complete molecular response, *MMR* Major molecular response, *HCC* Hepatocellular carcinoma, *od* Once a day, *e.o.d* Every other day, *PR* Partial response, *CR* Complete response, *EGFR* Epidermal growth factor receptor, *PFS* Progression-free survival, *OS* Overall survival, *LFT* Liver function test, *BSA* Body surface area, *IM* Imatinib, *FBC* Full blood count, *G* Grade

**Table 5:** Studies reporting other non-conventional dosing strategies

Publication and country	Aims	Design	Schedule	Reported outcomes		
				Efficacy	Toxicity	QoL
Tanvetyanon et al (2003) US [44]	To describe use of once-weekly imatinib due to cutaneous reactions	Case report, sample = 1, indication: ALL	Dose ranged from 300 mg od to 400 mg once a week, includes dose interruptions **Licensed monotherapy starting dose:600 mg od**	Nil	Rash, fever, and facial swelling, syncope (with daily dosing, resolved with weekly dosing)	Nil
Faber et al (2006) US [41]	To investigate safety and efficacy of intermittent dose imatinib following toxicities	Retrospective case series, sample = 12, indication: CML	Standard daily dose on initiation, changed to intermittent dose: range (300-600 mg 1-5 times a week) **Licensed monotherapy starting dose:400 mg od**	7 favourable cytogenetic responses (2 complete and 5 major). Improved cytogenetic response in 5 patients, and 1 haematological progression.	Malaise, fatigue, diarrhoea, fluid retention and muscle cramps.	Nil
Defina et al (2009) Italy [40]	To report tolerability and efficacy of alternate day dosing of lenalidomide	Prospective cohort study, sample = 6, indication: MDS	Lenalidomide 10 mg e.o.d (21/7 plus 7/7 break) **Licensed starting dose:10 mg od 21/7 plus 7/7 break**	Transfusion independence in all patients within 3-4 months. Cytogenetic response: CR (2) within 6-7 months, PR (3) within 7-9 months.	G3 thrombocytopenia (1), G3 neutropenia (2).	Nil
Satoh et al (2011) Japan [39]	To compare the efficacy of low-dose gefitinib vs. standard dose	Retrospective cohort sample = 114, indication: EGFR NSCLC	Standard dose group (S1) *n* = 61: 250 mg od. Reduced dose group (S2), *n* = 53: 250 mg e.o.d **Licensed monotherapy starting dose:250 mg od**	Non-inferiority (response and disease control) between groups. Response and disease control rates (83%, 98%) in S2 and (66%, 82%) in S1. Median PFS and 1-year PFS rate: S2 (11.8 months, 50%), S1 (9.9 months, 36%).	Nil	Nil
Hata et al (2012) Japan [42]	To describe efficacy and toxicity of intermittent erlotinib dosing in 4 cases	Case report, sample = 4, indication: EGFR NSCLC	Various doses used: 150 mg od, 150 mg e.o.d, 200 mg e.o.d, and 100 mg od. Different treatment durations and interruptions **Licensed monotherapy starting dose:150 mg od**	Responses ranged from partial response to progression.	(1) (2) and (3): G2–3 rash and paronychia resolved with 150 mg e.o.d in cases 1-3. G3 rash resolved with 100 mg od in case 4	Nil
Bojic et al (2012) Austria [43]	To report efficacy and toxicity of varying dosages of sunitinib used in 1 case	Case report, sample = 1, indication: metastatic RCC	Schedules used: 4/52 on 2/52 weeks off, continuous therapy, and 2/52 on 1/52 off **Licensed monotherapy starting dose:50 mg od 28/7 plus 2/52 break**	Different responses from different doses including CR, PR and relapse.	Fatigue and hypertension improved at 37.5 mg od. Improved toxicities and QoL at 2/52 on 1/52 off dosing	QoL improved
Popat et al (2014) UK [11]	To evaluate efficacy and cost-saving of alternate day dosing of lenalidomide	Prospective cohort study, sample = 39, indication: multiple myeloma	Starting dose 25 mg od for 21/28 followed by 1/52 break. Dose reduced to e.o.d at 25 mg, 15 mg, or 10 mg per dose. Median duration 12 cycles **Licensed starting dose:25 mg od 21/7 plus 7/7 break**	ORR 85%, CR 3%, VGPR 23%, PR 59%, MR 13%, SD 0%, PD < 1%. Median PFS 11.5 months, Median OS 36.5 months. Cost-savings: £19,408.43/patient	Nil	Nil
Abdel-Wahab et al (2014) US [45]	To report efficacy and toxicity of intermittent vemurafenib used in 1 case	Case report, sample = 1, indication: BRAF-mutant melanoma	Vemurafenib dose: range 480-960 mg bd, with interruptions due to toxicity. Cobimetinib added at 9 months: dose range 40-60mg od **Licensed monotherapy dose:960 mg bd**	Marked disease improvement at week 2. Near CR on intermittent schedule.	High WCC, fatigue, anaemia	Nil
Fukuizumi et al (2015) Japan [46]	To describe management of crizotinib with dose reductions in 1 case	Case report, sample = 1, indication: ALK positive NSCLC	Dose variation: 250 mg bd, 250 mg od, 250 mg every 3 days, and 250 mg bd every 3 days, with dose interruptions. **Licensed monotherapy starting dose:250 mg bd**	Significant tumor response on escalating to 250 mg bd. Significant response maintained for 13 months with 250 mg bd every 3 days.	Dyspnoea, chest discomfort.	Nil
Tsukita et al (2015) Japan [47]	To report a case of oesophagitis resolved with alternate day crizotinib	Case report, sample = 1, indication: ALK positive NSCLC	Dose variation: 250 mg bd, 200 mg bd, 200 mg e.o.d, 250 mg bd e.o.d, with interruptions due to toxicity **Licensed monotherapy starting dose:250 mg bd**	Shrinkage of the spinal metastases then re-growth.	Dysphasia and retrosternal pain, severe oesophagitis, Grade 3 ALT elevation.	Nil
Saponara et al (2016) Italy [48]	To report four cases treated with low dose imatinib	Case report, sample = 4, indication: GIST	Dose ranges: 800 mg od to 300 mg od, with interruptions **Licensed monotherapy starting dose:400 mg od**	Responses variable from good to stable disease.	Fatigue, periorbital and leg oedema, and skin toxicities.	Nil

Licensed doses of individual drugs have been listed in bold, following schedule information, for reader's information

*Abbreviations*: *ALL* Acute lymphocytic leukaemia, *CML* Chronic myeloid leukaemia, *od* Once a day, *e.o.d* Every other day, *bd* Twice a day, *MDS* Myelodysplastic syndrome, *CR* Complete response, *PR* Partial response, *EGFR* Epidermal growth factor receptor, *NSCLC* Non-small cell lung cancer, *RCC* Renal cell carcinoma, *ORR* Overall response rate, *VGPR* Very good partial response, *MR* Minor response, *SD* Stable disease, *PD* Progressive disease, *PFS* Progression-free-survival, *OS* Overall survival, *WCC* White cell count, *ALK* Anaplastic lymphoma kinase, *GIST* Gastrointestinal stromal tumor, *ALT* Alanine aminotransferase

### Quality appraisal

For dose interruption studies, clinical trials were appraised as moderate to high [21, 22], moderate [23] and moderate to low [20]. Cohort studies were moderate to high [24], moderate [25–27] and moderate to low [28–31]. Case reports were moderate [10, 32].

For dose reduction studies, cohort studies were appraised as moderate [18, 33] and moderate to low [34, 35]. The quality of case reports was moderate [17], moderate to low [19, 36, 37] and low [38].

For the remaining various dosing strategies, cohort studies were appraised as moderate [11, 39], and moderate to low [40]. Case reports were moderate [41–43] and moderate to low [44–48].

### Non-standard dosing strategies

#### Dose interruptions

Three clinical trials reported findings from non-standard dose interruptions (Table 3). A small randomised trial (*n* = 23) conducted by Mangiacavalli and colleagues (2012) investigated efficacy and adverse effects of a one-week interruption of thalidomide following daily administration for 3 weeks, compared to continuous therapy [21]. The study reported a trend for worse overall survival (OS, *p* < 0.001) and progression free survival (PFS, *p* = 0.02) in the intermittent arm compared to the continuous arm, with no difference in peripheral neuropathy; however patient numbers in this study were very small (≤ *n* = 30), which prevented this trial from obtaining definitive efficacy data [21]. Mangiacavalli and colleagues (2012) highlighted the place of this dosing strategy in patients experiencing toxicity (peripheral neuropathy), but recommended that a balance needs to be maintained with the desired efficacy outcomes [21].

Dose interruption was also not supported by findings from a single-arm, non-randomised recurrent glioma trial (*n* = 90, of which *n* = 64 had glioblastoma) [20]. The standard cycle 1 dose of temozolomide monotherapy for the treatment of glioma is 150 mg/m^2^ once a day for 5 days (days 1-5) of every 28 days cycle [2]. This trial investigated an alternative schedule (days 1-7, and days 15-21 of a 28 days cycle, i.e. 1-week-on/ 1-week-off) [20]. PFS rate in glioblastoma group was 43.8% at 6 months, median PFS was 24 weeks [20]. OS rate at 12 months was 23%, median OS was 38 weeks [20]. Toxicity outcomes were reported, but QoL outcomes were not [20]. Data from this trial suggest that the alternating weekly schedule of temozolomide showed clinically meaningful improvement in survival outcomes compared to the registration trial (PFS rate at 6 months: 21%) [20]. Wick and colleagues (2007) argued that the alternating-weekly schedule is feasible, safe, and effective and recommended further investigation of this strategy in randomised studies [20].

Russo and colleagues (2015) conducted a single arm, open-label trial, in which they investigated the use of 1 month on/1 month off schedule of imatinib in 96 CML patients aged ≥65 years [23]. Although this trial did not report toxicity or QoL outcomes, there were no transformations (progressions) of CML to an accelerated or blast phase of disease using this alternative schedule [23]. However, 16 patients lost complete cytogenetic response (CCgR) and molecular response (MR3.0) and 16 patients lost MR3.0 alone [23]. In optimal and stable responders, Russo and colleagues (2015) suggest that because all the patients who relapsed could be brought back to optimal response, a policy of intermittent imatinib treatment is feasible, successful in about 50% of patients, and safe [23].

Dose interruption strategies were also reported in one clinical trial and eight retrospective cohort studies, which investigated the use of non-standard schedules of sunitinib in renal cell carcinoma (RCC) (Table 3). Jonasch and colleagues (2018) conducted a small (*n* = 59) phase II single arm open label study of sunitinib in previously untreated RCC [22]. Patients were started on 50 mg 2 weeks on and 1 week off, and were eligible for further dose/schedule reductions (Level − 1 to − 5) [22]. The primary endpoint was < 15%  ≥G3 fatigue, diarrhoea or hand-foot syndrome (HFS) [22]. The latter was not met because 25% experienced one of those  ≥G3 toxicities [22]. Jonasch and colleagues (2018) described how their primary end point of decreased grade 3 toxicity was not met; however treatment with this modified schedule is associated with reduced grade 4 toxicity, a low patient discontinuation rate, and high efficacy [22].

Most of the sunitinib cohort studies compared standard dosing of (4 weeks on and 2 weeks off), to (2 weeks on and 1 week off), except for one study, which did not detail the non-standard dose and schedule (reported as any dosing schedule different to standard) [29]. Due to variance in reported outcomes in these cohort studies, it was not possible to conduct meta-analysis on this data; however key findings are summarised as follows.

The sample size of sunitinib cohort studies ranged widely from 8 to 460 participants (mean = 150). Reported efficacy outcomes included, complete response (CR), partial response (PR), overall response rate (ORR), OS, PFS; in addition to toxicity. QoL outcomes were only reported in two studies. Where reported, participants receiving alternative dose interruption schedules showed either similar or improved responses and survival outcomes, and fewer or equivalent high grade toxicities, compared to standard schedule. Overall, authors of the eight retrospective studies recommend that intermittent dosing should be further investigated in prospective studies to confirm its safety and efficacy.

Vemurafenib dose interruption was reported in two case reports [10, 32]. One report of one case described a number of dosing levels employed [32], and the other case report described dosing levels variable from one patient to another (*n* = 6), and a range of reponses (from good to disease relapse) [10]. Dooley and colleagues (2014) recommend that in clinical practice, intermittent dosing should be considered as an alternative to dose reduction/termination in the management of vemurafenib toxicity [10].

#### Dose reductions

Four cohort studies retrospectively evaluated dose reductions of either imatinib to treat CML, gefitinib or erlotinib to treat NSCLC [18, 33–35]. Clinical efficacy outcomes were reported as one or more of the following: time to progression (TTP), PFS OS, in addition to toxicities. QoL was not reported for any of the cohorts. None of these studies reported all outcomes (i.e. efficacy, toxicity, and QoL).

Breccia and colleagues (2010) reported findings that could not be easily interpreted for the purpose of this review, because OS was not compared between the different imatinib doses used [18]. In addition, Jung Sung and colleagues (2014) did not report sufficient detail about imatinib dose reductions received to be able to fully analyse findings in this review [33]. Neither imatinib study referenced above reported QoL or sufficient toxicity outcomes (i.e. none reported by Breccia et al., and only toxicities leading to dose reductions reported by Jung Sung et al). Breccia and colleagues (2010) recommend that longer follow-up and further observation of a larger cohort of CML patients are required to establish the safety and the long-term responses to dose reduction of imatinib [18]. From their findings, Jung Sung and colleagues (2014) suggest that imatinib dose adjustments that take into account body surface area (BSA), could improve the clinical outcomes in patients with chronic phase CML [33]; but like other authors of studies reviewed herein, recommend that further prospective studies are required [33].

A large cohort study comparing standard dose (*n* = 240) to dose-reduced (*n* = 23) gefitinib showed improved median OS and PFS in the dose-reduced subgroup [35]. The study only reported toxicities leading to dose reductions. Similarly, Binder and colleagues (2010) compared standard dose of erlotinib (*n* = 31) to two dose reduction groups (*n* = 9; n = 9) in patients with NSCLC and reported TTP, but no survival outcomes [34]. Patient numbers were unequal between the standard dose subgroup and dose reduction subgroups. Although, Hoon Sim and colleagues (2014) recommend further investigation of dose reduction of gefitinib in a prospective trial [35], Binder and colleagues (2010) did not report a clear recommendation for or against erlotinib dose reduction for the treatment of NSCLC [34].

Dose reduction strategies were also reported in five case studies (Table 4) that described the use of reduced doses of imatinib or dasatinib for CML [17, 19, 37], and sorafenib for advanced hepatocellular cancer (HCC) [36, 38]. Serpa and colleagues (2010) and Jamison and colleagues (2016) suggested that low-dose dasatinib therapy in intolerant patients may be tried before drug discontinuation [17, 37] or a change is considered [37]. In addition, Zipin and colleagues (2014) recommended to conduct a dose reduction trial in this patient cohort [19]. Shinoda and colleagues (2015) recommended that sorafenib dose reduction described in their report should be further explored [36].

#### Other dosing strategies

Three cohort studies investigated other dosing strategies including alternate-day dosing of gefitinib for NSCLC [39], lenalidomide for myelodysplastic syndrome (MDS) and multiple myeloma (MM) [11, 40] (Table 5).

Lenalidomide studies were single arm cohorts, although the MM study was prospective [11]. All three studies had small sample sizes. Alternate dosing of gefitinib was found to have non-inferiority in response and disease control rates compared to standard [39]. Toxicity outcomes were only reported in the MM study. QoL were not reported for any of the three studies. Authors of the two alternate dosing of lenalidomide recommend either for this schedule to be explored in a larger cohort of patients [40], or for its clinical outcomes to be confirmed in prospective studies [11]. Satoh and colleagues (2011) identify the specific needs to test non-standard gefitinib dosing schedules in frail patients who are at risk of treatment toxicity [39].

Eight case studies reported a variety of alternative dosing strategies (Table 5). These small studies described non-standard dosing practices in patients receiving either imatinib for leukaemia or gastrointestinal stromal tumour (GIST) [41, 44, 48], crizotinib or eroltinib for NSCLC [42, 46, 47], vemurafenib for malignant melanoma [45], and sunitinib for RCC [43]. Some of the evaluated strategies included combinations of intermittent dosing, and various dose reductions in response to experienced toxicities. For example, Faber and colleagues (2006) used imatinib in CML at doses ranging from to 300 mg to 600 mg, and frequencies ranging from one to five times a week in 12 patients, rather than a daily dose. This was considered a plausible treatment option for patients with persistent myelotoxicity [41] Other authors additionally suggested that non-standard dosing strategies may help to individualise treatment to reduce toxicities [46, 47], maintain QoL and support patient compliance [43].

## Discussion

This review aimed to systematically identify evidence of, and outcomes (efficacy, toxicity, QoL) from, non-standard dosing of oral SACT in oncology and malignant haematology, in order to inform prescribing practices. This review identified a wide range of study types: clinical trials, prospective and retrospective cohort studies as well case reports/series. Included studies ranged across both solid tumours (two thirds of all included studies) and malignant haematology (one third). The amount and quality of reported outcomes depended considerably on the study design. Efficacy/survival outcomes were reported in most studies. Varying toxicity outcomes were reported in cohort studies and case reports. QoL outcomes were not reported in the majority of studies. In order to inform current prescribing practice, this review focused on categorising common non-standard dosing interventions. The secondary aim of this review, which was to inform research evaluating the feasibility of oral SACT non- standard dosing practice, has been partially met by indicating some non-standard dosing strategies that warrant further investigation in large-scale randomised controlled trials.

Our recommendations for non-standard dosing strategies based on the evidence reviewed herein are as follows:

- Drug interruption strategy

The benefit of dose interruption was dependent on the individual drug, with some studies showing no benefit. Although data reported in the temozolomide single arm non-randomised trial does not provide statistically significant evidence to implement its dose interruption strategy [20], the intervention does warrant further investigation in a large randomised controlled trial. This recommendation is in line with recommendations of study authors.

Results from the imatinib (1 month on/off) trial do not draw definitive conclusions that intermittent treatment can be offered to optimal and stable responders [23]. The findings, however, indicate a role for alternative treatment schedules tailored to individual patients, particularly those experiencing significant toxicities, in agreement with study authors.

Drawbacks of the sunitinib dose interruption trial in RCC were small patient number, single arm design, and lack of detailed reporting of PFS, OS and toxicities outcomes for levels − 1 to − 5 dose reductions [22]. QoL outcomes were not reported [22]. In addition, the trial did not meet its primary endpoint of < 15%  ≥G3 toxicities using alternative schedule but equally it did not compare this schedule to standard dosing schedule [22].Therefore, this small phase II trial does not provide sufficient evidence to issue a generalised recommendation to employ sunitinib dosed at 50 mg 2 weeks on and 1 week off, as alternative to standard dose. A larger scale randomised prospective study which compares this dosing strategy of sunitinib to its traditional dosing schedule is warranted in order to draw conclusions.

Evidence from the cohort studies that examined the use of dose-interrupted sunitinib for patients with RCC did suggest some benefit over standard dosing, so it might be considered as a strategy for reducing toxicity in patients prescribed sunitinib for RCC. In the absence of robust efficacy/toxicity/QoL outcomes data, however, evidence from these cohort studies is not sufficient to support the described sunitinib dosing schedules as alternative to standard dosing. We agree with the overall recommendation of others that this strategy warrants further investigation in a large prospective clinical trial to ensure efficacy, safety and improved patient-reported outcomes.

Although Dooley and colleagues (2014) recommended that in clinical practice intermittent dosing should be considered as an alternative to dose reduction/termination in the management of vemurafenib toxicity, we did not find sufficient evidence to issue a generalised recommendation to employ dose interruptions of vemurafenib in melanoma, based on the two case reports identified in this review [10, 32].

- Dose reduction strategy

The four cohort studies that retrospectively evaluated dose reductions of either imatinib to treat CML, gefitinib or erlotinib to treat NSCLC [18, 33–35] have a number of limitations, such as retrospective design, unequal patient numbers, and lack of reporting of toxicity and/or QoL outcomes. In agreement with authors of these cohort studies, it was not possible to draw conclusions about the impact of dose reduction based on the evidence reviewed in the above four cohort studies.

Out of the five case studies (Table 4) that described the use of reduced doses of imatinib or dasatinib for CML [17, 19, 37], and sorafenib for advanced hepatocellular cancer (HCC) [36, 38], very few studies reported toxicity or QoL outcomes. Although Serpa and colleagues (2010) and Jamison and colleagues (2016) suggested low-dose dasatinib therapy before treatment discontinuation due to toxicity [17, 37], based on efficacy and survival outcomes alone, we did not find sufficient evidence to support such dose reductions.

- Other dosing strategies

Evidence from the three cohort studies identified this review is not sufficient to support alternate day dosing of gefitinib or lenalidomide [11, 39, 40], but it calls for investigation in large scale randomised prospective clinical trials to compare it to standard dosing, in agreement with study authors. Alternate day dosing of lenalidomide is emerging in practice as a non-standard dosing strategy. However, there is currently no evidence from robust, randomised, large-scale studies assessing the efficacy, safety and QoL outcomes to support this practice routinely.

Overall, results from the case reports were inconclusive, primarily due to limitations in the design of the studies, small sample sizes and lack of detail in reporting toxicities and QoL outcomes. Our findings, therefore, differ from some case studies authors who suggest that modified schedules for imatinib, crizotinib and sunitinib can be used to manage toxicities.

In the UK, in view of the increasing cancer population and new available therapies, prescribing practice undertaken by physicians has been extended to non-medical prescribers (NMP) in the healthcare workforce to meet capacity demands. All prescribers in cancer clinics, including NMPs, need clear protocols, guidelines, and algorithms to support clinical decisions about safe, effective and in-context prescribing practice. Findings from this review are a reflection of increasing current practice of non-standard dosing of oral SACT.

Prescribers meet recurrent challenges of maintaining patients on life-saving cancer treatments, which carry varying risks from a wide spectrum of limiting toxicities. Intentional non-adherence and patient-controlled dosing (i.e. taking the drug only when patient feels able to) due to treatment toxicity has been reported and can result in diminished extent of clinical benefit from therapy [49], and sub-optimal prospects of the overall treatment pathway. It is, therefore, imperative that clinical trials take into account real-life, intention to treat data when analysing the efficacy of licenced drugs, so that protocols and guidelines support safe and efficacious practice.

Supportive care, depending on toxicity of a specific drug, is used to treat acute toxicities e.g. topical products to prevent or treat cutaneous toxicity from erlotinib [50] or to speed up recovery e.g. use of granulocyte-colony stimulating factor in patients treated with lenalidomide to stimulate neutrophil production [51]. In addition, for first generation oral SACT (e.g. imatinib), physicians tend to use their clinical judgment based on experience of prescribing the drug to apply alternative dosing schedules to manage toxicities and maintain a disease response on an individual basis.

For newer generation oral SACTs, adjusting the dose of oral SACT to manage toxicities usually follows recommendations from the Summary of Product Characteristics (SPC), but depends on the practice of the individual prescriber. Strategies can include dose-interruption until toxicity reduces or totally resolves, dose-reduction or in cases with high-grade toxicity treatment discontinuation. In the case of the UK, funding for novel agents by NHS England (NHSE) is in place where prescribing follows evidence, NICE recommendations and Cancer Drugs Fund (CDF) criteria [52]. Use of unlicensed oral SACT dosing strategies is, therefore, not funded.

The number of licensed oral SACT evaluated in this review was 78 [2]. Licensing of newer agents, such as new oral kinase inhibitors and T-cell checkpoint inhibitor immunotherapy, will inevitably change the way some cancers, such as renal cell carcinoma, are currently treated. It is important, however, to acknowledge the likelihood of both the ongoing use of current oral SACT and an increase in non-standard dosing strategies to manage toxicities, improve QoL, and ultimately maintain patients taking these agents in the longer-term.

This review reports findings from studies that describe and evaluate alternative prescribing strategies for sunitinib. These strategies suggest a role for dose-interruption strategies using this drug to treat RCC, but large randomised controlled trials are needed to determine statistically significant, clinically meaningful results about treatment responses (OS and PFS), toxicities and QoL. Studies are also needed to explore how non-standard dosing of oral SCT, such as dose interruption, might affect treatment adherence.

Dose-reduced imatinib in CML can be explored as an option particularly in older patients with major cytogenetic or molecular responses. Dose reductions of other agents such as gefitinib, crizotinib and sorafenib are not supported by findings of this review. Prescribers might choose to use dose reduction for individual patients to support continuation of treatment prior to cessation due to toxicities, as reported in the imatinib in CML trial and sunitinib in RCC cohort studies.

Due to the very high cost of oral SACT, future non-standard dosing studies should include health economics and utility analysis. Use of dose interruptions or dose-reduction suggests a cost-saving, because fewer doses are prescribed and administered, and reduced costs can result from these toxicity management strategies. This does, however, need to be balanced with the potential outcome to treatment, and the need for an evidence base for these alternative strategies to confirm their efficacy, toxicity and QoL profiles.

### Strengths and limitations

No previous systematic review has explored the practice of non-standard dosing of oral SACT. To ensure transparency and to facilitate scrutiny of this review, a systematic protocol was registered and published prior to conducting the review, which was undertaken according to best practice and reporting guidelines. Each stage of the review process was independently double-screened and any discrepancies discussed among the research team until agreement was reached. There was no date limitation imposed on the review, so studies were selected on the basis of prescribing practices that were relevant to current practice. One limitation of the search strategy was restricting the search to publications in English; however the search expansion strategy ensured a comprehensive and sensitive review.

One of the challenges of this study was reviewing evidence generated from a diverse range of study designs and variety of tumour-types treated with different oral SACTs. Although this constrained the ability to conduct a meta-analysis, retrieving a breadth of literature was deemed necessary to fully scope non-standard dosing practices in the treatment of oncological and haematological tumours. We chose to analyse findings of the review by type of non-standard prescribing strategy, due to the limited number of studies published about any one drug, with the exception of sunitinib. It is possible that analysing the data from any single drug used for a specific tumour type might provide more robust recommendations; however we would argue that currently the data set is not sufficiently large to conduct this type of analysis, which is a limitation of this review.

The quality of evidence reviewed was limited by the small sample size of many studies, baseline characteristics not being reported or recorded, use of retrospective study designs, lack of measurement of toxicity and/or QoL outcomes, and some dose-reduction studies not reporting the reduced dose administered. Studies were also un-blinded, which possibly could have been blinded.

Lack of baseline measurements meant it was difficult to assess whether there was any bias due to multiple variables between treatment groups. There was also lack of detail in reporting toxicity outcomes in some studies. Given the justification for using non-standard dosing is to alleviate toxicity, we consider that fully measuring toxicity and quality of life outcomes is a fundamental requirement when investigating non-standard dosing strategies. There was also an absence of health economics and utility analysis, except for one study [11].

## Conclusions

There is limited evidence to support current non-standard prescribing practices. There is an indication that dose interruption might be a safe and efficacious strategy to reduce treatment toxicity for patients prescribed sunitinib for RCC. This strategy might also have a role in other tumour groups and other types of oral SACT; however there is a need for large-scale, ideally blinded, prospective, RCTs that measure OS, PFS, toxicity outcomes, QoL outcomes and health utilities to be conducted.

## Additional file

Additional file 1: **Table S1.** List of oral SACT included in the search strategy. (DOCX 16 kb)

## Abbreviations

AE: Adverse events
ALT: Alanine aminotransferase
BNF: British National Formulary
BSA: Body surface area
CASP: Critical Appraisal Skills Programme
CCyR: Complete cytogenetic response
CDF: Cancer Drugs Fund
CHF: Congestive heart failure
CINAHL: Cumulative Index to Nursing and Allied Health Literature
CML: Chronic myeloid leukaemia
E.O.D: Every other day
GIST: Gastrointestinal stromal tumour
HCC: Hepatocellular carcinoma
IM: Intramuscular
IT: Intrathecal
IV: Intravenous
MCyR: Major cytogenetic response
MDS: Myelodysplastic syndrome
MESH: Medical Subject Heading
MHRA: Medicines and Healthcare Products Regulatory Agency
MM: Multiple myeloma
MR: Minor response
NHS: National Healthcare Service
NHSE: NHS England
NSCLC: Non-small cell lung cancer
OS: Overall survival
PD: Progressive disease
PFS: Progression free survival
PN: Peripheral neuropathy
PR: Partial response
PRISMA-P: Preferred Reporting Items for Systematic Review and Meta-Analysis Protocols
QoL: Quality of life
RCC: Renal cell carcinoma
SACT: Systemic anticancer therapy
SC: Subcutaneous
SD: Stable disease
TOT: Time on treatment
TTF: Time to treatment failure
TTP: Time to progression

## References

[1] Mansinho A (2017). The future of oncology therapeutics. Expert Rev Anticancer Ther.

[2] British Medical Association (2017). Royal Pharmaceutical Society. British national formulary.

[3] Electronic Medicines Compendium. Summary of Product Characteristics: Glivec 400 mg film-coated tablets. Available from: https://www.medicines.org.uk/emc/product/5566 (Accessed 12th Feb 2018).

[4] Electronic Medicines Compendium. Summary of Product Characteristics: SUTENT 50 mg hard capsules. Available from: https://www.medicines.org.uk/emc/product/7966 (Accessed 12.02.2018).

[5] Patridge A (2002). Adherence to Therapy With Oral Antineoplastic Agents. J Natl Cancer Inst.

[6] Huang WC (2016). Medication adherence to oral anticancer drugs: systematic review. Expert Rev Anticancer Ther.

[7] Electronic Medicines Compendium. Summary of Product Characteristics: Zelboraf 240 mg Film-coated Tablets. Available from: http://www.medicines.org.uk/emc/medicine/26056 (Accessed 12.02.2018).

[8] National Institute for Health and Care Excellence. NICE Guidance. Available from: https://www.nice.org.uk/guidance (Accessed 12.02.2018).

[9] National Institute for Health and Care Excellence. Technology appraisal guidance. Available from: https://www.nice.org.uk/about/what-we-do/our-programmes/nice-guidance/nice-technology-appraisal-guidance (Accessed 12.02.2018).

[10] Dooley AJ, Gupta A, Bhattacharyya M, Middleton MR (2014). Intermittent dosing with vemurafenib in BRAF V600E-mutant melanoma: review of a case series. Ther Adv Med Oncol.

[11] Popat R, Khan I, Dickson J, Cheesman S, Smith D, D’Sa S, Rabin N, Yong K (2015). An alternative dosing strategy of lenalidomide for patients with relapsed multiple myeloma. Br J Haematol.

[12] Grant MJ, Booth A (2009). A typology of reviews: an analysis of 14 review types and associated methodologies. Health Inf Libr J.

[13] Moher D, Shamseer L, Clarke M, Ghersi D, Liberati A, Petticrew M (2015). Preferred reporting items for systematic review and meta-analysis protocols (PRISMA-P) 2015 statement. Syst Rev.

[14] Djebbari F, Stoner N, Lavender V (2017). Non-conventional dosing of oral anticancer agents in oncology and malignant haematology: a systematic review protocol. Sys Rvs Journal.

[15] Critical Appraisal Skills Programme (CASP). CASP Appraisal Checklists. Available from: https://casp-uk.net/casp-tools-checklists/ (Accessed 12.02.2018).

[16] Manoharan A (1991). Management of myelofibrosis with intermittent hydroxyurea. Br J Haematol.

[17] Serpa M, Sanabani SS, Bendit I, Seguro F, Xavier F, Barroso CB, Conchon M, Dorlhiac-Llacer PE (2010). Efficacy and Tolerability after Unusually Low Doses of Dasatinib in Chronic Myeloid Leukemia Patients Intolerant to Standard-Dose Dasatinib Therapy. Clin Med Insights: Oncol.

[18] Breccia M, Cannella L, Stefanizzi C, Latagliata R, Nanni M, Diverio D, Santopietro M, Federico V, Alimena G (2010). Cytogenetic and molecular responses in chronic phase chronic myeloid leukaemia patients receiving low dose of imatinib for intolerance to standard dose. Hematol Oncol.

[19] Zipin D, Savage D (2004). Reduced dose Imatinib Mesylate therapy for chronic myeloid leukemia. Leuk Lymphoma.

[20] Wick A, Felsberg J, Steinbach JP, Herrlinger U, Platten M, Blaschke B, Meyermann R, Reifenberger G, Weller M, Wick W (2007). Efficacy and Tolerability of Temozolomide in an Alternating Weekly Regimen in Patients With Recurrent Glioma. J Clin Oncol.

[21] Mangiacavalli S, Albani G, Caravita T, Cocito F, Pascutto C, Zappasodi P, Bringhen S, Palumbo A, Cazzola M, Corso A (2012). Similar neurotoxicity of an alternating compared to a continuous low-dose schedule of thalidomide for relapsed/refractory multiple myeloma. Leuk Lymphoma.

[22] Jonasch E, Slack RS, Geynisman DM, Hasanov E, Milowsky MI, Kimryn Rathmell W, Stovall S, Juarez D, Gilchrist TR, Pruitt L, Ornstein MC, Plimack ER, Tannir NM, Rini BI (2018). Phase II Study of Two Weeks on, One Week off Sunitinib Scheduling in Patients With Metastatic Renal Cell Carcinoma. J Clin Oncol.

[23] Russo D, Malagola M, Skert C, Cancelli V, Turri D, Pregno P, Bergamaschi M, Fogli M, Testoni N, De Vivo A, Castagnetti F, Pungolino E, Stagno F, Breccia M, Martino B, Intermesoli T, Cambrin GR, Nicolini G, Abruzzese E, Tiribelli M, Bigazzi C, Usala E, Russo S, Russo-Rossi A, Lunghi M, Bocchia M, D’Emilio A, Santini V, Girasoli M, Di Lorenzo R, Bernardi S, Di Palma A, Cesana BM, Soverini S, Martinelli G, Rosti G, Baccarani M (2015). Managing chronic myeloid leukaemia in the elderly with intermittent imatinib treatment. Blood Cancer J.

[24] Pan X, Huang H, Huang Y, Liu B, Cui X, Gan S, Ye J, Xu D, Lu C, Zhou Q, Lin L, Hong Y (2015). Sunitinib dosing schedule 2/1 improves tolerability, efficacy, and health-related quality of life in Chinese patients with metastatic renal cell carcinoma. Urol Oncol: seminars and original investigations.

[25] Bradley J, Atkinson SK, Wang X, Bathala T, Corn P, Tannir NM, Jonasch E (2014). Clinical outcomes for patients with metastatic renal cell carcinoma treated with alternative Sunitinib schedules. J Urol.

[26] Kondo T, Takagi T, Kobayashi H, Iizuka J, Nozaki T, Hashimoto Y, Ikezawa E, Yoshida K, Omae K, Tanabe K (2014). Superior Tolerability of Altered Dosing Schedule of Sunitinib with 2-Weeks-on and 1-Week-off in Patients with Metastatic Renal Cell Carcinoma—Comparison to Standard Dosing Schedule of 4-Weeks-on and 2-Weeks-off. Jpn J Clin Oncol.

[27] Bracarda S, Iacovelli R, Boni L, Rizzo M, Derosa L, Rossi M, Galli L, Procopio G, Sisani M, Longo F, Santoni M, Morelli F, Di Lorenzo G, Altavilla A, Porta C, Camerini A, Escudier B (2015). Sunitinib administered on 2/1 schedule in patients with metastatic renal cell carcinoma: the RAINBOW analysis. Ann Oncol.

[28] Buti S, Donini M, Lazzarelli S, Passalacqua R (2012). A new modified schedule of Sunitinib for metastatic renal cell carcinoma: a retrospective analysis. Acta Biomed.

[29] Neri B, Vannini A, Brugia M, Muto A, Rangan S, Rediti M, Tassi R, Cerullo C (2013). Biweekly sunitinib regimen reduces toxicity and retains efficacy in metastatic renal cell carcinoma: a single-center experience with 31 patients. Int J Urol.

[30] Ohzeki T, Fukasawa S, Komaru A, Namekawa T, Sato Y, Takagi K, Kobayashi M, Uemura H, Ichikawa T, Ueda T (2014). Efficacy of traditional and alternative sunitinib treatment schedules in Japanese patients with metastatic renal cell carcinoma. Int J Urol.

[31] Miyake H, Harada K-i, Miyazaki A, Fujisawa M (2015). Improved health-related quality of life of patients with metastatic renal cell carcinoma treated with a 2 weeks on and 1 week off schedule of sunitinib. Med Oncol.

[32] Koop I, Stazger MA, Kapp A, Auschild A, Gutzmer R (2014). Intermittent BRAF-inhibitor therapy is a feasible option: report of a patient with metastatic melanoma. Br JDermatol.

[33] Sung HJ, Lee SR, Choi IK, Park Y, Choi CW, Kim H-J, Yhim H-Y, Kim BS (2015). Imatinib Mesylate Dose Adjustment Based on Body Surface Area for CML Chronic Phase Patients Intolerant to Standard Dosage. Acta Haematol.

[34] Binder D, Buckendahl A-C, Hubner R-H, Schlattmann P, Temmesfeld-Wollbruck B, Beinert T, Suttorp N (2012). Erlotinib in patients with advanced non-small-cell lung cancer: impact of dose reductions and a novel surrogate marker. Med Oncol.

[35] Sim SH, Keam B, Kim D-W, Kim TM, Lee S-H, Chung DH, Heo DS (2014). The gefitinib dose reduction on survival outcomes in epidermal growth factor receptor mutant non-small cell lung cancer. J Cancer Res Clin Oncol.

[36] Shinoda M, Kishida N, Itano O, Ei S, Ueno A, Kitago M, Abe Y, Hibi T, Yagi H, Masugi Y, Tanabe M, Aiura K, Sakamaoto M, Tanimoto A, Kitagawa Y (2015). Long-term complete response of advanced hepatocellular carcinoma treated with multidisciplinary therapy including reduced dose of sorafenib: case report and review of the literature. World J Surg Oncol.

[37] Jamison C, Nelson D, Eren M, Gauchan D, Ramaekers R, Norvell M, Copur MS (2016). What is the optimal dose and schedule for Dasatinib in chronic myeloid leukemia: two case reports and review of the literature. Oncol Res.

[38] Abbadessa G, Rimassa L, Pressiani T, Carrillo-Infante C, Cucchi E, Santoro A (2011). Optimized management of advanced hepatocellular carcinoma: four long-lasting responses to sorafenib. World J Gastroenterol.

[39] Satoh H, Inoue A, Kobayashi K, Maemondo M, Oizumi S, Isobe AG, Saijo Y, Yoshizawa H, Hagiwara K, Nukiwa T (2011). Low-dose Gefitinib treatment for patients with advanced non-small cell lung Cancer harboring sensitive epidermal growth factor receptor mutations. J Thorac Oncol.

[40] Defina M, Rondoni M, Gozzetti A, Aprile L, Chitarrelli I, Fabbri A, Lauria F, Bocchia M (2010). Lenalidomide on alternative days is effective in myelodysplastic syndrome with 5q- deletion. Br J Haematol.

[41] Faber E, Naušová J, Jarošová M, Egorin MJ, Holzerová M, Rožmanová Š, Marešová I, Divoký V, Indrák K (2006). Intermittent dosage of imatinib mesylate in CML patients with a history of significant hematologic toxicity after standard dosing. Leuk Lymphoma.

[42] Hata A, Fujita S, Kaji R, Nanjo S, Katakami N (2013). Dose Reduction or Intermittent Administration of Erlotinib: Which Is Better for Patients Suffering from Intolerable Toxicities?. Intern Med.

[43] Bojic M, Schmidinger M (2012). Dose matters: the importance of appropriate dosing: a case report on Sunitinib treatment in a patient with metastatic renal cell carcinoma. Clin Genitourin Cancer.

[44] Tanvetyanon T, Nand S (2003). Overcoming recurrent cutaneous reactions from Imatinib using once-weekly dosing. Ann Pharmacother.

[45] Abdel-Wahab O, Klimek VM, Gaskell AA, Viale A, Cheng D, Kim E, Rampal R, Bluth M, Harding JJ, Callahan MK, Merghoub T, Berger MF, Solit DB, Rosen N, Levine RL, Chapman PB (2014). Efficacy of Intermittent Combined RAF and MEK Inhibition in a Patient with Concurrent BRAF- and NRAS-Mutant Malignancies. Cancer Discov.

[46] Fukuizumi A, Miyanaga A, Seike M, Kato Y, Nakamichi S, Chubachi K, Matsumoto M, Noro R, Minegishi Y, Kunugi S, Kubota K, Gemma A (2015). Effective Crizotinib schedule for an elderly patient with ALK rearranged non-small-cell lung cancer:a case report. BMC Res Notes.

[47] Tsukita Y, Fukuhara T, Kobayashi M, Morita M, Suzuki A, Watanabe K, Noguchi T, Kurata Y, Suno M, Maemondo M (2015). Alternate-day Treatment with Crizotinib for Drug-induced Esophagitis and Liver Damage in a Patient with EML4-ALK Fusion Gene-positive Lung Adenocarcinoma. Intern Med.

[48] Saponara M, Gatto L, Di Nunno V, Tabacchi E, Fanti S, Di Scioscio V, Nannini M, Gruppioni E, Altimari A, Fiorentino M, Santini D, Ceccarelli C, Zompatori M, Biasco G, Pantaleoa MA (2016). Successful treatment with personalized dosage of imatinib in elderly patients with gastrointestinal stromal tumors. Anti-Cancer Drugs.

[49] Lea Ann Hansen. Impact of Nonadherence to Cancer Therapy. Journal of Haematology Oncology Pharmacy. Online First. Available at: http://www.jhoponline.com/jhop-issue-archive/2015-issues/june-vol-5-no-2?view=article&secid=11003:top-sec-11003&artid=14873:top-14873 (accessed 05.04.2018).

[50] Electronic Medicines Compendium. Summary of Product Characteristics: Tarceva 150 mg film-coated tablets. Available from: https://www.medicines.org.uk/emc/product/8845 (Accessed 12th Feb 2018).

[51] Electronic Medicines Compendium. Summary of Product Characteristics: Revlimid. Available from: https://www.medicines.org.uk/emc/product/347. (Accessed 12th Feb 2018).

[52] NHS England. Cancer Drugs Fund. Available from: https://www.england.nhs.uk/cancer/cdf/. (Accessed 06.04.2018).

