# Supplementary material for: A systematic review of non-standard dosing of oral anticancer therapies
Source: BMC Cancer. 2018 Nov 22;18:1154. doi: 10.1186/s12885-018-5066-2 (PMC6249819; doi:10.1186/s12885-018-5066-2)
Supplement: Supplementary file 1 — Table S1. List of oral SACT included in the search strategy. (DOCX 16 kb) [file 12885_2018_5066_MOESM1_ESM.docx]

**Table S1**

| **Drug names** | | | | |
| --- | --- | --- | --- | --- |
| 1. imatinib Mesylate | 17. thioguanine | 33. everolimus | 49.temsirolimus | 65. enzalutamide |
| 2. bexarotene | 18. topotecan | 34. gefitinib | 50. trametinib | 66. flutamide |
| 3. busulfan | 19. treosulfan | 35. ibrutinib | 51. vandetanib | 67.medroxyprogesterone |
| 4. capecitabine | 20. tretinoin | 36. idelalisib | 52.vemurafenib | 68. megestrol |
| 5. chlorambucil | 21. trifluridine | 37. lapatinib | 53. vismodegib | 69. norethisterone |
| 6. cyclophosphamide | 22. vinorelbine | 38. nilotinib | 54.thalidomide | 70. diethylstilbestrol |
| 7. estramustine | 23. afatinib | 39. nintedanib | 55. lenalidomide | 71. ethinylestradiol |
| 8. etoposide | 24. axitinib | 40. olaparib | 56.pomalidomide | 72. idarubicin |
| 9. hydroxycarbamide | 25. bosutinib | 41. osimertinib | 57. anastrozole | 73. methotrexate |
| 10. melphalan | 26. cabozantinib | 42.panobinostat | 58. exemestane | 74. trifluridine |
| 11. mercaptopurine | 27. cobimetinib | 43. pazopanib | 59. letrozole | 75. lenvatinib |
| 12. lomustine | 28. crizotinib | 44. ponatinib | 60. Tamoxifen | 76. venetoclax |
| 13. mitotane | 29. ceritinib | 45. regorafenib | 61. Toremifene | 77. ixazomib |
| 14. procarbazine | 30. dabrafenib | 46. ruxolitinib | 62. Abiraterone | 78. palbociclib |
| 15. tegafur | 31. dasatinib | 47. sorafenib | 63. bicalutamide |  |
| 16. temozolomide | 32. erlotinib | 48. sunitinib | 64. cyproterone |  |

**Table S1: List of oral SACT included in the search strategy**
